# Lack of Association between Fluconazole Susceptibility and *ERG11* Nucleotide Polymorphisms in *Cryptococcus neoformans* Clinical Isolates from Uganda

**DOI:** 10.3390/jof8050508

**Published:** 2022-05-15

**Authors:** Priscilla Belbir Atim, David B. Meya, Elliot S. Gerlach, Dennis Muhanguzi, Allan Male, Benedict Kanamwanji, Kirsten Nielsen

**Affiliations:** 1 Infectious Diseases Institute, Kampala P.O. Box 22418, Uganda; priscillaatim47@yahoo.co.uk; 2 Department of Microbiology and Immunology, University of Minnesota, Minneapolis, MN 55455, USA; gerl0060@umn.edu (E.S.G.); knielsen@umn.edu (K.N.); 3 College of Veterinary Medicine Animal Resources and Biosecurity, Makerere University, Kampala P.O. Box 7062, Uganda; dennis.muhanguzi@mak.ac.ug; 4 International Centre for Tropical Agriculture (CIAT)—Uganda, Kampala P.O. Box 6247, Uganda; a.m.ssekamate@cgiar.org; 5 National Microbiology Reference Laboratory (NMRL), Kampala P.O. Box 7272, Uganda; bkanamwanji@gmail.com

**Keywords:** *ERG11*, *Cryptococcus neoformans*, single nucleotide polymorphisms, fluconazole, IC_50_, prophylaxis, cryptococcal meningitis

## Abstract

Fluconazole is the drug of choice for cryptococcal meningitis (CM) monoprophylaxis in resource-limited settings such as Uganda. Emerging fluconazole resistance linked to mutations in the *Cryptococcus neoformans* *ERG11* gene (CYP51) has been observed in clinical isolates. Currently, the single nucleotide polymorphisms [SNPs] in the *Cryptococcus* spp. *ERG11* gene that could be responsible for fluconazole resistance are poorly characterized within Ugandan *C. neoformans* clinical isolates. If available, this information would be useful in the management of cryptococcosis among HIV patients. This cross-sectional study investigates the SNPs present in the coding region of the *C. neoformans* *ERG11* gene to determine the relationship between the SNPs identified and fluconazole susceptibility of the clinical isolates. 310 *C. neoformans* isolates recovered from the Cerebrospinal Fluid (CSF) of patients with HIV and cryptococcal meningitis were examined. The fluconazole half-maximal inhibitory concentrations (IC_50_ range: 0.25–32 μg/mL) was determined using the microbroth dilution method. A total of 56.1% of the isolates had low IC_50_ values of <8 μg/mL while 43.9% had high IC_50_ values ≥ 8 μg/mL. We amplified and sequenced 600 bp of the *ERG11* coding sequence from 40 of the clinical isolates. Novel synonymous and 2 missense mutations, S460T and A457V, were identified in the *ERG11* gene. The identified SNPs were not associated with differences in fluconazole IC_50_ values in vitro (*p* = 0.179).

## 1. Introduction

Fluconazole is a major prophylaxis used both before and during antiretroviral treatment against cryptococcosis [1]. The recommended antifungal treatment for acute cryptococcal meningitis (CM) is a combination of amphotericin B with flucytosine for the induction treatment phase, followed by fluconazole for the consolidation and maintenance treatment phases [2]. In resource-limited settings, such as in sub-Saharan Africa, fluconazole is a commonly available drug that is used for induction monotherapy [3]; however, is not recommended due to poor survival rates, slow fungal clearance and the emergence of fluconazole resistance [3,4].

Despite the adoption of rigorous approaches towards managing cryptococcosis among HIV patients, HIV-related *Cryptococcus neoformans* mortality is estimated at 2412 persons per year in Uganda [5] due to probable antifungal resistance and late diagnosis [6,7]. An increase in the basal fluconazole MIC has been observed in Ugandan clinical isolates that is associated with the widespread use of fluconazole in HIV patients [8]. Similarly, in South Africa, a two-fold increase in fluconazole minimum inhibitory concentrations (MICs) was observed in clinical *Cryptococcus* spp. isolates over a decade [9]. Thus, the emergence of fluconazole-resistant *Cryptococcus* spp. isolates poses a challenge for the effective management of cryptococcal infections [8,9]. Currently, there are no interpretive breakpoints for in vitro antifungal susceptibility testing of *C. neoformans* and thus it is difficult to define phenotypes associated with fluconazole susceptibility and resistance [10]. Instead, clinical isolates are typically described as having low or high IC_50_ values [11].

Fluconazole inhibits ergosterol synthesis by interfering with the 14-α lanosterol demethylase enzyme *ERG11* [12]. Importantly, the mechanisms of fluconazole resistance in *Cryptococcus* spp. have been linked to increased or decreased expression of the *ERG11* gene [13], aneuploidy [14,15], overexpression of the membrane efflux pump proteins [16] and mutations in the *ERG11* gene. These *ERG11* gene alterations affect the ability of the drug to bind to the Erg11p protein [17,18]. An increase in the fluconazole MIC was also observed in *C. neoformans* clinical isolates with mutations at the fluconazole binding site on the Erg11p protein [19]. The specific mutations in Erg11p that were identified among fluconazole-resistant *C. neoformans* isolates include G484S, G470R, Y145 F, and I99V [18,19,20]. Factors that have exacerbated the emergence of fluconazole resistance include increased clinical use of fluconazole [8] and widespread use of triazole fungicides [21].

In this study, SNPs in the *ERG11* gene of clinical *C. neoformans* isolates from HIV-infected individuals in Uganda were investigated to identify possible genetic changes that are associated with the observed increasing fluconazole MICs in Uganda. These datasets are important to characterize SNPs in the *ERG11* gene in the context of the fluconazole susceptibility of *C. neoformans*.

## 2. Materials and Methods

The *C. neoformans* clinical isolates used in this study were collected as part of the ASTRO (Adjunctive Sertraline for the Treatment of HIV-Associated Cryptococcal Meningitis) clinical trials [22,23,24] and were obtained from individuals with HIV and cryptococcal meningitis co-infections. The isolates were obtained from cerebrospinal fluid (CSF) and stored in a 20% glycerol solution at −80 °C in the Department of Medical Microbiology at Makerere University. The clinical isolates were cultured from the glycerol stocks onto Sabouraud Dextrose Agar (SDA) plates (Difco, Sparks, MD, USA). The extracted DNA samples were stored at −20 °C until use. The clinical *C. neoformans* cultures were either shipped as glycerol stocks or the culture was placed on filter paper and shipped at room temperature to the University of Minnesota, where they were subsequently stored as −80 °C glycerol stocks.

### 2.1. Fluconazole Minimum Inhibitory Concentration (MIC) Broth Microdilution Assays

The Fluconazole IC_50_ values of 310 *C. neoformans* isolates were determined using the EUCAST microbroth dilution assay, as described in [25]. Briefly, the *C. neoformans* clinical isolates were plated onto yeast-peptone-dextrose (YPD) plates containing 0.10 mg/mL chloramphenicol and incubated at 30 °C for 48 h. Overnight cultures were then prepared in YPD broth containing 10 μg/mL chloramphenicol and incubated at 30 °C with shaking. The resulting cultures were centrifuged and washed 3 times with sterile water, resuspended, and a 1:100 dilution was prepared for cell quantification using a hemocytometer. The final inoculum of each isolate for the microbroth dilution MIC assay was prepared to EUCAST specifications in sterile water. A 50 mg/mL stock solution of fluconazole (Sigma-Aldrich, St. Louis, MO, USA) was prepared in DMSO. Fluconazole test concentrations ranged from 0.25–128 μg/mL as described by the EUCAST microbroth dilution assay [25].

All of the broth microdilution assays were carried out using a 2% glucose RPMI-1640 medium (Sigma R-8755, St. Louis, MO, USA) with a final inoculum concentration of 0.5 × 10^5^–2.5 × 10^5^ [25]. Immediately after inoculation, the optical density was measured at 600 nm (OD600) using a Biotek Synergy H1 Hybrid reader (Winooski, VT, USA). Plates were then incubated 72 h at 37 °C, and a second OD600 measurement was taken. The IC_50_ for each strain was determined based on analysis of the well turbidity measurements, using the OD600, as described in [8]. A KN99α reference strain [26], with a known fluconazole IC_50_ of 2 μg/mL was included as an inter-assay calibration reference in every MIC plate to verify accuracy across all of tthe MIC plates. IC_50_ was defined as the first fluconazole drug concentration at which ≥50% of the growth was inhibited [11].

### 2.2. DNA Extraction, Amplification, and Sequencing

Single colony isolates were plated on SDA for 40 of the *C. neoformans* clinical isolates. DNA was extracted from 3 independent single colonies for each of the 40 isolates. The colonies were suspended in 150 µL of 1X TE buffer (10 mM Tris, pH 8.0, 1 mM EDTA, and pH 8.0) in a 1.5 mL microcentrifuge tube and vortexed for 2 min. Thereafter, the suspension was heated in a microwave for 2 min, cooled to room temperature and centrifuged at 13,000 rpm for 2 min. The supernatant was then transferred to a fresh 0.5 mL tube from which 2 µL was used as amplicon in a 25 µL PCR reaction.

A 600 bp fragment within the *ERG11* gene-coding region, centered on the known G484 SNP site, was amplified using a single pair of primers ERGF-5′-AGTTGCCCATCATGGACTCTA-3′ and ERGR-5′-GAAGACTTACACGGTAATTGG-3′ in a final PCR volume of 25 µL. The amplification reactions were performed using an Eppendorf Mastercycler Thermal Cycler (Eppendorf AG, Hamburg, Germany). The PCR reaction contained 1X PCR buffer, 1U Taq DNA polymerase (New England BioLabs, Ipswich, MA, USA), 0.5 µM of each primer, 0.5 µM dNTPs (New England BioLabs, Ipswich, MA), and 2 µL DNA. The amplification program was as follows: initial denaturation at 95 °C for 5 min followed by 35 cycles, each consisting of denaturation at 94 °C for 20 s, annealing at 50 °C for 30 s, and extension at 72 °C for 1 min. The program ended with a final extension step at 72 °C for 10 min. The amplicons were resolved on a 1.6 % agarose gel at 90 V for 1 h in 1X TBE buffer (0.045 M Tris-borate and 1 mM EDTA, pH 8.2). The gel was soaked in 0.5 µg/mL ethidium bromide for 20 min to stain and imaged using a Syngene G: BOX gel documentation system (Fredrick, MD, USA).

Exonuclease 1-Shrimp Alkaline Phosphatase (ExoSAP-IT) (Applied Biosystems, Vilnius, Lithuania) was used to clean-up the PCR products for sequencing. ExoSAP-IT (Applied Biosystems, Vilnius, Lithuania) was diluted (1:3) in PCR grade water. The cleanup reaction was comprised of 2 µL of diluted ExoSAP-IT and 3.5 µL of PCR product. The cleanup reaction was performed using a SimpliAmpTM Thermal Cycler (Applied Biosystems). The program for cleanup was 37 °C for 45 min then 80 °C for 15 min. The cleanup products were stored at −20 °C until sequencing.

The sequencing reaction mixture contained 1 µL BigDyeTM terminator (Applied Biosystems), 1.5 µL 5X buffer (Applied Biosystems), 1 µL 10µM of either the ERGF or ERGR primer, 1 µL ExoSAP-IT treated PCR product, and 5.5 µL PCR grade water (10 µL in total). The cycle sequencing reaction was performed using a SimpliAmpTM Thermal Cycler (Applied Biosystems) using the following program: 25 cycles each consisting of denaturation at 96 °C for 10 s, annealing at 50 °C for 5 s, and extension at 60 °C for 4 min. The gene products were then sequenced using the Sanger method [27] on an ABI 3730 automated DNA sequencer (Applied Biosystems). The generated sequences were analyzed using Sequencing Analysis v.5.3 software (Applied Biosystems). The *C. neoformans* partial coding region *ERG11* gene nucleotide sequences were each entered into the BLASTn [28] sequence analysis program on NCBI and SNPs were identified. A graphical representation of the nucleotide changes in the coding region of the *ERG11* gene was generated using Weblogo version 2.8.2 [29].The exon sequences were then analyzed using AUGUSTUS for protein prediction [30]. The subsequent protein fragments were aligned to the clinical *C. neoformans* reference strain INM 972624, NCBI accession ID AAP12370.1, using the Multiple Sequence Comparison by Log-Expectation (MUSCLE) [31] software package and amino acid changes were identified in the multiple sequence alignment. All of the sequences from this study were deposited in the NCBI GenBank with accession number IDs MZ673051-MZ673090.

### 2.3. Analysis

To compare the association of the SNPs and the Fluconazole IC_50_ values and human mortality, a linear regression analysis was performed, using STATA SE 15 (StataCorp LLC, College Station, TX, USA)software.

## 3. Results

### 3.1. In Vitro Fluconazole Susceptibility

This study analyzed 310 isolates with fluconazole IC_50_ values ranging from low values of <8 μg/mL (n = 174) to high IC_50_ values of 8 μg/mL(n = 93), 16 μg/mL(n = 39) and 64 μg/mL(n = 4) (Table 1). The geometric mean for the isolates was 5.1 μg/mL. Recommended fluconazole breakpoints for *C. neoformans* are not defined yet and as such, we considered IC_50_ < 8 μg/mL low, and IC_50_ ≥ 8 μg/mL high [11].

A 600 bp DNA fragment was amplified and sequenced from the region of the *ERG11* gene coding region that has previously been shown to be a “hot spot” for SNPs Sequencing was performed on a subset of 40 clinical isolates, derived from 37 patients Table 2 provides a summary of the characteristics of the 37 patients from whom the isolates were taken. 24.3% (9/37) of the patients died from CM and 8.1% (3/37) of the patients experienced a CM relapse.

### 3.2. SNP Analysis of the ERG11 Gene

**Figure 1:**
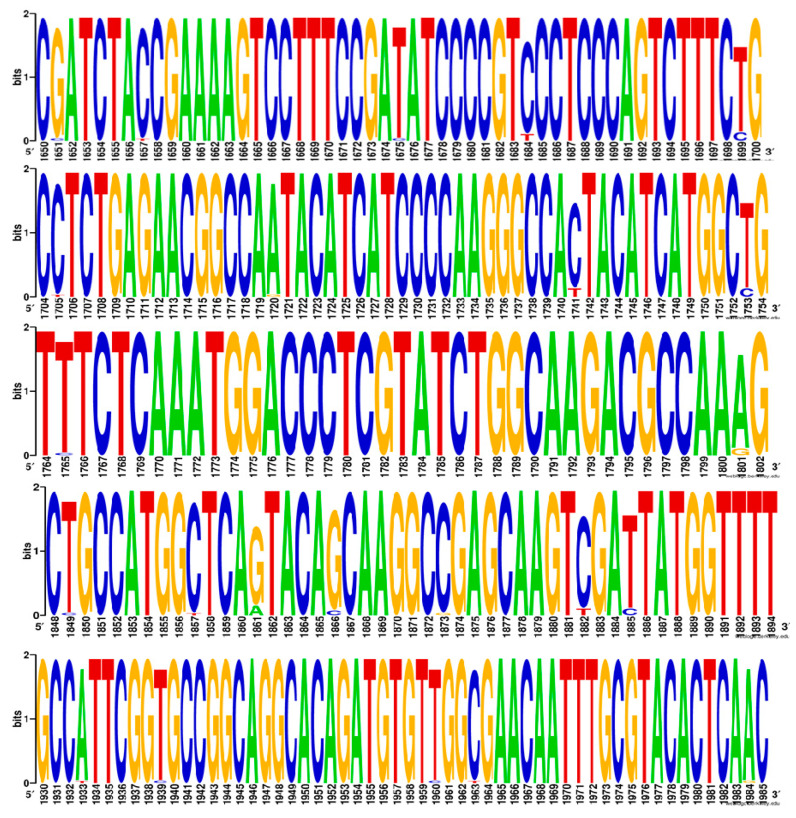
Sequence logo showing the SNPs within the *C. neoformans ERG11* gene.

SNPs found within the *ERG11* coding region are presented in Table 3 and their relative abundance within the population is shown in Figure 1. The largest number of SNPs were found in *C. neoformans* clinical isolate 11420 (MZ673090) (Table 3).

The synonymous polymorphism A1861G was present in all the clinical isolate sequences except isolate numbers 110414 (MZ673086), 110414 D3 (MZ673087), 110399 (MZ673089), and 110420 (MZ673090). Two non-synonymous SNPs (indicated with a ^‡^ in Table 3) resulted in the amino acid changes A457V and S460T. Novel synonymous SNPs found in the *C. neoformans* clinical isolates that had not been previously reported were also identified. Surprisingly, low fluconazole IC_50_s < 8 μg/mL were observed in isolates with amino acid changes; and isolates with high fluconazole IC_50_s did not contain *ERG11* SNPs (*p* = 0.179, −1.37 *t*-test).

## 4. Discussion

The goal of the present study was to determine the fluconazole susceptibility of *C. neoformans* clinical isolates from a Ugandan patient cohort and investigate the presence of SNPs in a highly conserved coding region of the *ERG11* gene. The *C. neoformans* isolates in this study were classified as having either low IC_50_ values (56.1%) or high IC_50_ values (43.9%) based on the classification recommended by Gerlach et al. [11]. The high incidence of fluconazole IC_50_ values ≥ 8 µg/mL in this group of *C. neoformans* clinical isolates is consistent with surveillance data in sub-Saharan Africa and elsewhere over the past two decades, with an increase in MICs across many geographical regions [9,32,33,34]. This increased resistance poses a public health challenge, especially in Africa where fluconazole is widely prescribed and frequently used as a monotherapy for both consolidation and maintenance CM therapy [9]. In addition, the increasing trend of high fluconazole IC_50_ values observed among *C. neoformans* clinical isolates in Uganda [8,11] underscores the need to review the current recommended fluconazole dosages for optimal therapeutic outcomes [33]. Studies have recommended 800 mg/day for consolidation therapy of patients infected with isolates that have high MICs as a mechanism to improve clinical outcomes [33]. While there are no standardized breakpoints for *C. neoformans*, IC_50_s ≥ 8 μg/mL have been associated with poor clinical outcomes [10,35].

The subset of 40 sequenced isolates, which represents 13% of the entire population, was representative of the fluconazole susceptibility and patient outcomes observed in the larger 310 ASTRO clinical trial isolate set [22,23,24]. Additionally, we showed that this subset readily contained isolates with polymorphisms in *ERG11* but no fluconazole sensitivity. Based on our observation of multiple isolates with polymorphisms that were not linked with high fluconazole IC_50_s, the subset we analyzed is sufficiently large to show the necessary diversity. In the subset of patients for which we preformed *ERG11* sequencing of their clinical isolate, there was a 24.3% (9/37) CM mortality rate. Moreover, 77. 8% (7/9) of the clinical isolates from the patients who died had low fluconazole IC_50_ of <8 μg/mL, while only 22.2% (2/9) had a high fluconazole IC_50_ of ≥8 μg/mL. Although 8.1% (3/37) of the patients were relapse cases, only 1 of these patients had an IC_50_ of ≥8 μg/mL.

The disparity between the overall clinical outcomes in patients with low in vitro fluconazole IC_50_ has been consistently observed across studies and has been attributed to possible antifungal drug tolerance [36]. Tolerance in fungi is defined as slow growth of a subpopulation of cells at drug concentrations above the IC_50_, with this growth often observed after longer incubation periods beyond those used for the standard MIC assays [36]. However, the characterization of fluconazole tolerance in *Cryptococcus* spp. remains poorly defined. Fluconazole tolerance may be underestimated when MICs are performed in 2% glucose due to the ability of *Cryptococcus* spp. to exhibit in vivo fluconazole tolerance in the low glucose host environment [37,38]. In addition, host factors such as patient drug adherence, pharmacokinetic data [4] and host-specific immune reconstitution inflammatory syndrome [39] can collectively cause treatment failure.

**Figure 2:**
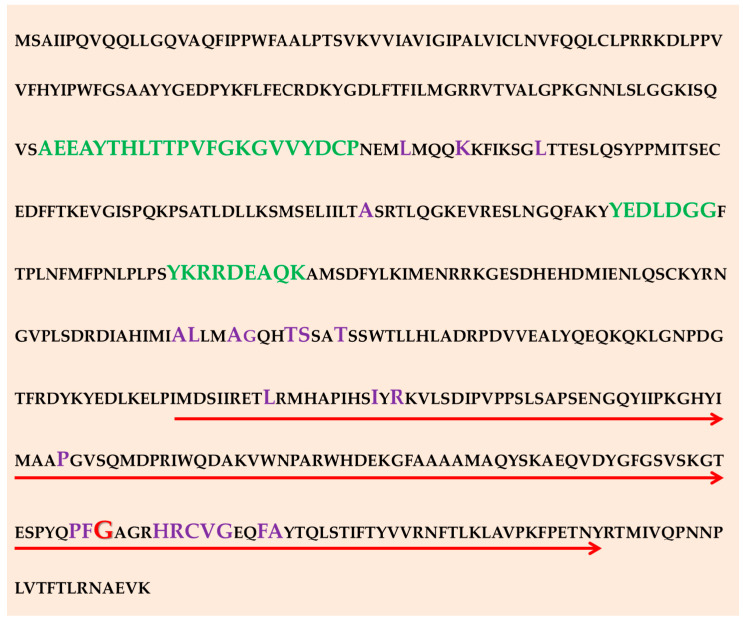
Amino acid sequence of the *C. neorfomans ERG11* gene coding sequence indicating the region sequenced in this study. Thered arrows show the gene region which was sequenced that covers the “hotspot” region at position 484 (indicated red) previously associated with azole resistance [44]. Heme binding sites are highlighted in purple [20]. Substrate recognition sites are highlighted in green [45].

We observed 2 amino acid changes (A457V, S460T), along with other synonymous nucleotide changes, in the *ERG11* gene coding region. These differences in sequence could be due to natural variations in the *ERG11* genetic code. However, sequence analysis of the Ergl1p protein across different fungi has previously shown that the enzyme ligand-binding pocket site amino acid domains are highly conserved (Figure 2) [40]. This conservation is most likely required for the integrity and protein function of the 14α-demethylase activity of the protein. [41]. Polymorphisms in the *ERG11* gene, with or without resulting amino acid changes have previously been described as possible mechanism for fluconazole resistance among clinical isolates of *C. neoformans* [42]. The presence of these polymorphisms suggests there is genetic diversity within *C. neoformans* and may highlight allelic variations in the *ERG11* gene. *ERG11* gene SNPs have been observed in other yeasts of medical importance, such as the *Candida* spp. [43]. It is unknown whether synonymous polymorphisms, such as those we observed in our population, are directly causing fluconazole resistance. Another possibility is that the accumulation of these nucleotide polymorphisms and mutations, coupled with other factors such as recombination and selective environmental pressure, may indirectly affect Erg11p function [41].

For example, the missense mutation S460T that we observed was also previously observed in fluconazole-resistant *C. neoformans* isolates [18]. Although specific *C. neoformans ERG11* gene amino acid mutations are known to cause high fluconazole MICs [17,20], our study showed that the S460T and A457V mutations were not linked to increased fluconazole IC_50_ in our clinical isolates. Based on this observation, we conclude that the *Cryptococcus ERG11* gene is polymorphic and the SNPs we identified in our study are not the main cause of the high fluconazole IC_50_ observed in our and previous studies.

This study had several limitations. First, our *ERG11* SNP investigation was limited to 600 bp of the gene coding region. Analysis of the entire ERG11 gene region, including the promoter, could yield additional data and identify SNPs outside of the region we analyzed that associate with drug resistance. Second, the patient clinical data (CD4 count) collected in the parent ASTRO clinical trial had missing data. Specifically, only 19/37 (51.3%) of the patient CD4 counts were collected at the time of CSF culture collection. In addition, while the clinical trial did not exclude patients that had or were receiving HIV therapy, the current status of that therapy was not provided with the isolates. Ultimately, Cryptococcus-related HIV fatalities remain high in sub-Saharan Africa. Emerging fluconazole resistance is a major public health challenge and effective antifungal therapy is critical [9]. Yet this study suggests that this increased resistance may not be linked to SNPs within the *C.neoformans* “hot spot” region of the Erg11p protein [44]. Other mechanisms of resistance, such as aneuploidy (heteroresistance) and over-expression of *ERG11* or ABC fluconazole transporter genes, may be critically important in *C. neoformans* fluconazole resistance and need to be examined in clinical isolate cohorts. Until then, management of CM needs to incorporate development of new non-azole drugs as well as combination therapy approaches that utilize drugs with different modes of action [11].

## 5. Conclusions

This study revealed nonsynonymous polymorphisms and a novel synonymous polymorphism in the *ERG11* gene-coding region from clinical isolates of *C. neoformans.* Our results suggest that these SNPs are not associated with the high fluconazole IC_50_ observed in some of the isolates. Larger studies involving more clinical isolates and genome-wide association studies on these isolates is needed to investigate the genetic variations within the high and low fluconazole IC_50_ isolates. Future studies of the virulence of isolates with high and low fluconazole IC_50_ should be performed in animal models of cryptococcosis to determine the association between fluconazole IC_50_ and isolate virulence potential. In addition, studies on the functional impact of SNPs on *ERG11* gene expression, along with alternative molecular mechanisms for the increasing fluconazole resistance, such as intrinsic heteroresistance and over-expression of the ABC fluconazole transporter genes, should be performed.

## Figures and Tables

**Table 1:** *C. neoformans* clinical Isolates Fluconazole IC_50_.

	Low IC_50_					High IC_50_			
Fluconazole IC_50_ (μg/mL)	0.25	0.5	1	2	4	8	16	32	64
N [proportion]	0 (0)	7 (2.2)	16 (5.16)	44 (14.2)	107 (34.5)	93 (30)	39 (12.6)	0 (0)	4 (1.29)

**Table 2:** Characteristics of the 37 study patients whose isolates were selected for *ERG11* sequencing.

Parameter	Results n = 37
Age in years: median (range)	35.9 (20–65)
Gender	
Male	21 (56.7%)
Female	16 (43.2%)
CM Relapse	
History of CM	3 (8.1%)
No History of CM	34 (91.9%)
Mortality	
Alive	28 (75.7%)
Dead	9 (24.3%)

**Table 3:** Single nucleotide polymorphisms (SNPs) in the partial *ERG11* gene-coding region from 40 *C. neoformans* clinical isolates show no association with fluconazole IC_50_ values.

Isolate	SNP Type	SNP	IC_50_ (μg/mL)	GenBank Codes
110159	A1861G	Synonymous	8	MZ673063
110166	A1861G	Synonymous	8	MZ673065
110174	A1861G	Synonymous	16	MZ673067
110180	C1741T	Synonymous	8	MZ673055
110183	A1861G	Synonymous	2	MZ673066
110242	A1861G	Synonymous	4	MZ673077
110246	A1861G	Synonymous	8	MZ673064
110252	A1861G	Synonymous	0.5	MZ673070
110271	A1861G	Synonymous	2	MZ673058
110288	C1741T, A1861G	Synonymous	2	MZ673080
110290	C1741T, A1861G	Synonymous	4	MZ673084
110301	A1861G	Synonymous	8	MZ673071
110352	A1861G	Synonymous	4	MZ673060
110353	A1861G	Synonymous	4	MZ673083
110355	A1861G	Synonymous	8	MZ673074
110389	A1861G	Synonymous	8	MZ673059
110390	A1861G	Synonymous	2	MZ673052
110395	A1861G	Synonymous	2	MZ673078
110399	C1684T, T1699C, T1753C, A1801G, C1882T, ^‡^ G1866C	**Missense mutation S460T ^1^**	4	MZ673089
110404	A1861G	Synonymous	4	MZ673054
110413	A1861G	Synonymous	8	MZ673081
110414 D1	C1684T, T1699C, T1753C, C1882T, T1885C, ^‡^ G1866C	**Missense mutation S460T ^1^**	2	MZ673086
110414 D3	C1684T, T1699C, T1753C, A1801G, C1882T, T1885C,	Synonymous	2	MZ673087
110416	A1861G	Synonymous	8	MZ673056
110418	A1861G	Synonymous	4	MZ673079
110420	G1651C, C1657T, T1675C, T1699C, C1705T, A1720G, C1741T, T1753C, T1765C, T1768Y, A1801G, T1849C, ^‡^ C1857T, C1873G, T1903C, A1933T, T1939C, T1960C, C1963T, A1984C	**Missense mutation A457V ^1^**	4	MZ673090
110422	A1861G	Synonymous	4	MZ673068
110428	-	Identical to reference wild-type *C. neoforman* sequence AY265353.1	4	MZ673075
110429	A1861G	Synonymous	4	MZ673085
110433	A1861G	Synonymous	8	MZ673076
110433 *	A1861G	Synonymous	4	MZ673057
110435	A1861G	Synonymous	4	MZ673073
110439	A1861G	Synonymous	4	MZ673082
110441	A1861G	Synonymous	4	MZ673062
110444 D1	A1861G	Synonymous	4	MZ673061
110444 D7	A1861G	Synonymous	8	MZ673051
110449	-	Identical to reference wild-type *C.neoforman* sequence AY265353.1	1	MZ673088
110450	A1861G	Synonymous	8	MZ673053
110451	A1861G	Synonymous	4	MZ673072
110461	A1861G	Synonymous	8	MZ673069

^‡^ Indicates non-synonymous mutations that change the amino acid sequence of Erg11p. ^1^ Isolates with mutations that resulted in amino acid changes had low fluconazole IC_50_ < 8 μg/mL. *Additional isolate from the same patient with identical SNP but different IC_50_.

## Data Availability

*ERG11* gene sequences for the clinical isolates are deposited in GenBank under accession numbers MZ673051-MZ673090.
